# Mutations in Epigenetic Modifiers in Myeloid Malignancies and the Prospect of Novel Epigenetic-Targeted Therapy

**DOI:** 10.1155/2012/469592

**Published:** 2011-06-26

**Authors:** Amir T. Fathi, Omar Abdel-Wahab

**Affiliations:** ^1^Department of Medicine, Massachusetts General Hospital, Boston, MA 02114, USA; ^2^Human Oncology and Pathogenesis Program and Leukemia Service, Memorial Sloan-Kettering Cancer Center, New York, NY 10065, USA

## Abstract

In the recent years, the discovery of a series of mutations in patients with myeloid malignancies has provided insight into the pathogenesis of myelodysplastic syndromes (MDSs), myeloproliferative neoplasms (MPNs), and acute myeloid leukemia (AML). Among these alterations have been mutations in genes, such as *IDH1/2*, *TET2*, *DNMT3A*, and *EZH2*, which appear to affect DNA and/or histone lysine methylation. Large clinical correlative studies are beginning to decipher the clinical importance, prevalence, and potential prognostic significance of these mutations. Additionally, burgeoning insight into the role of epigenetics in the pathogenesis of myeloid malignancies has prompted increased interest in development of novel therapies which target DNA and histone posttranslational modifications. DNA demethylating agents have been demonstrated to be clinically active in a subset of patients with MDS and AML and are used extensively. However, newer, more specific agents which alter DNA and histone modification are under preclinical study and development and are likely to expand our therapeutic options for these diseases in the near future. Here, we review the current understanding of the clinical importance of these newly discovered mutations in AML and MDS patients. We also discuss exciting developments in DNA methyltransferase inhibitor strategies and the prospect of novel histone lysine methyltransferase inhibitors.

## 1. Introduction

The increasing use of systematic genome-wide discovery efforts in patients with a variety of myeloid malignancies has led to the rapid discovery of a series of recurrent genetic abnormalities underlying these disorders. Remarkably, a large number of these alterations appear to be in genes whose function is known, or suspected, to be involved in epigenetic regulation of gene transcription. In the last 3 years, alone mutations in the genes *TET2*, *IDH1*, *IDH2*, *DNMT3a*, and *EZH2* have all been found in patients with myeloproliferative neoplasms (MPNs), myelodysplastic syndromes (MDSs), and/or acute myeloid leukemia (AML). Although the functional implications of these mutations and how precisely they contribute to abnormal hematopoiesis and leukemogenesis is being heavily investigated and not yet clarified, a number of potentially clinically important implications of these mutations may already be apparent. First, mutations in several of these genes likely hold prognostic importance for patients, and these genetic alterations, thereby, may serve as prognostic markers for risk stratification and aid in therapeutic decision making. Secondly, mutations in several of these genes may specifically impact DNA methylation and/or histone posttranslational modifications in such a manner that is therapeutically targetable. Mutations in several of these genes, such as *IDH1* and *IDH2*, have been proven to result in a gain-of-enzymatic function which holds the prospect for development of novel targeted therapeutics. This review focuses on the clinical relevance of recently discovered epigenetic alterations in patients with myeloid malignancies and the prospect for novel targeted therapeutics against aberrant epigenomic characteristics in patients with MDS and AML.

## 2. Recently Identified Mutations in Epigenetic Modifiers in Myeloid Malignancies

### 2.1. *IDH1* and *IDH2* Mutations

Genome sequencing of AML has recently led to the discovery of mutations in the genes encoding isocitrate dehydrogenase (*IDH1 and IDH2*). IDH1 is a key cytosolic enzyme in the Krebs cycle. It catalyzes the decarboxylation of isocitrate to *α* keto-glutarate (*α*-KG), leading to the production of nicotinamide adenine dinucleotide phosphate (NAD-P). The isocitrate dehydrogenase 2 (*IDH2*) gene encodes a homologous protein which catalyzes the same reaction in mitochondria. IDH mutations have been extensively studied and are frequently found alterations in low-grade gliomas. They have also been discovered in a small subset of the highly aggressive glioblastomas, where they confer a more favorable prognosis [1–6]. 

*IDH* mutations were subsequently identified in AML and other myeloid malignancies, including MDS and MPNs [7, 8]. All discovered *IDH* mutations reside in the active site of the enzyme and participate in isocitrate binding [9]. They are missense alterations affecting arginine-132 (R132) in IDH1, and either the analogous arginine residue (R172), or the arginine-140 (R140) residue in the IDH2 protein [7, 10–15]. The common recurrence of *IDH* mutations in AML suggests an importance in leukemogenesis. All mutations are missense and heterozygous, suggesting that the alterations lead to a “gain of function” [9]. It has been shown that the mutant forms of IDH cannot catalyze the conversion of isocitrate to *α*-KG [16]. Dang et al. reported that the mutated R132H IDH1, in place of the normal process of isocitrate decarboxylation, catalyzes an NADPH-dependent reduction of *α*-KG to 2-hydroxyglutarate (2-HG). 2-HG is a metabolite which is normally present at very low levels in cells [17], 2-HG levels have been found to be elevated in *IDH*-mutant glioma samples, and Gross et al. reported that IDH1 R132 mutations also lead to production and accumulation of 2-HG in AML blasts, greater than 50-fold higher than their nonmutant counterparts. Elevated 2-HG levels in IDH-WT samples led to the first discovery of IDH2 mutations, which accounted for elevated 2-HG levels in these AML cells. The elevation in 2-HG levels has also been noted in sera of patients with *IDH*-mutant AML [9].

Studies of *IDH* mutations in gliomas have suggested that they are an early event in the pathogenic process [5]. Their exact mechanism in leukemogenesis of AML is uncertain. Normal IDH function appears essential for normal cell growth and proliferation. IDH1 is one of only three cytosolic proteins which contribute to NADPH production which is essential for nucleotide and lipid synthesis. Ward et al. demonstrated that siRNA silencing of the IDH1 and IDH2 proteins led to a significantly reduced proliferative capacity [15]. Some investigators have suggested that accumulation of 2-HG plays an important role in this process, the “gain of function” neomorphic enzyme activity that promotes cancer [9, 18, 19]. Patients with a rare inherited condition called 2-hydroxyglutaric aciduria have elevated levels of 2-HG with an increased propensity for brain tumors. 2-HG has indeed been shown to increase reactive oxygen species in these patients [20, 21]. Additionally, 2-HG is homologous to *α*-KG in structure and thus may bind and interfere with essential enzymes that are activated by *α*-KG. Among these are prolyl hydroxylases which control the stability of and downregulate HIF-1*α* transcription factors, implicated in the pathogenesis of multiple malignancies [9, 19, 22].

**Figure 1:**
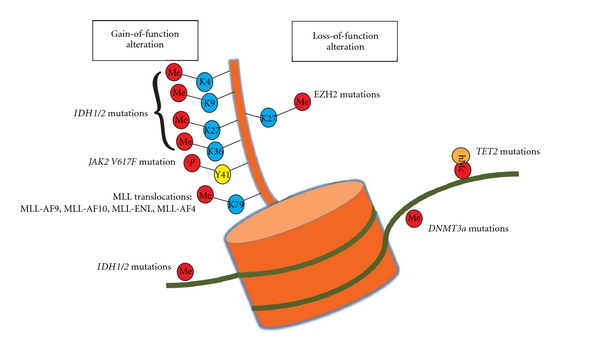
Specific histone and DNA posttranslational modifications shown to be associated with mutations in epigenetic modifiers in hematologic malignancies. Only genetic alterations which have some evidence for resulting in a gain or loss of function are displayed here. Mutations which result in the acquisition of hyperactivation or new enzymatic activity are displayed on the left of nucleosome while mutations which have evidence as resulting in a loss of enzymatic function are displayed on the right (translocations known to directly affect histone posttranslational modifications are listed in Table 1). The majority of mutations in epigenetic modifiers in myeloid malignancies recently identified are known to affect posttranslational modifications on the N-terminal tail of histone H3 or at cytosines of DNA as displayed here. Currently, the function of DNMT3a mutations in AML has yet to be extensively clarified, particularly the recurrent R882 heterozygous mutations.

The association of IDH mutations with aberrant hypermethylation has only recently been discovered. By studying samples from 398 AML patients in an Easter Cooperative Group (ECOG) E1900 trial, we found that IDH-mutant AML is associated with consistent and aberrant hypermethylation of various promoter sites involved in myeloid differentiation and leukemogenesis [23] (Figure 1). Promoter CpG sites are extremely important in the regulation of gene expression, specifically those of genes which mediate tumor suppression and differentiation, and DNA methylation can lead to transcriptional inactivation or chromosomal instability [24, 25]. Aberrant hypermethylation has been extensive described as a pathogenic process in forms of MDS and AML [26–29]. The discovery of aberrant hypermethylation and transcriptional inactivation of loci in relation to IDH-mutant AML is intriguing and significant. The potential mechanism for the hypermethylation and leukemogenesis in *IDH*-mutant disease may be related to downregulation of normal *α*-KG levels. Multiple enzymes are dependent on *α*-KG for their function, including the TET enzymes, which appear to play an important role in the differentiation of myeloid cells and promote demethylation by hydroxylating methylcytosine groups [30, 31]. 

Another recent study reported that altered 2-HG/*α*-KG levels present in *IDH1/2* mutant cells additionally results in the inhibition of a different set of *α*-KG-dependent enzymes, the Jumonji family of histone lysine demethylases (JHDM) [32]. There are 3 classes of enzymes which are known to antagonize histone methylation: (1) peptidylarginine deiminase, which removes methylarginine modifications to produce citrulline [33], (2) lysine specific demethylase 1 which removes H3K4me1/H3K9me1 marks in a reaction requiring flavin is a cofactor [34], and the Jumonji C domain family of histone demethylases which require iron Fe(II) and *α*-KG as cofactors. Unlike LSD1, which can only remove mono- and dimethyl lysine modifications, the JHDMs can remove methyl groups from all three histone methylation states. So far, JHDM family members have been shown to reverse the following lysine methyl marks: H3K36me1/2 (JHDM1) [35], H3K9me1/2 (JHDM2) [36], H3K9me2/3 (JHDM3) [35], and H3K36me3 (JHDM3) [35], H3K4me2/3 (JARID1) [37], and H3K27me2/3 (UTX/JMJD3) [38]. In addition, JMJD6 has been shown to encode an arginine-specific histone demethylase which demethylates H3R2me1/2 and H4R3me1/2 [39]. All of these marks may, therefore, be affected by the presence of *IDH1/2* mutations, and Xu et al. indeed demonstrated hypermethylation of many of these marks following introduction of *IDH1/2* mutations into cells [32] (Figure 1). These findings lend significant credence to the theory that hypermethylation of DNA and histone lysine/arginine modifications play a key role in the pathogenesis of AML in this subgroup of patients. Further work to delineate the complex epigenetic alterations to the transcriptional changes which promote leukemogenesis will be very enlightening.

Currently, our clinical use of genetics in AML for prognostication relies on (1) the use of cytogenetics to delineate patients into favorable, intermediate, or adverse cytogenetic categories and (2) molecular genotype of the genes *FLT3, NPM1 (nucleophosmin 1)*, and *CEBPA (CCAAT/enhancer-binding protein alpha*) for those patients with a normal karyotype. Multiple studies have demonstrated that patients with a normal karyotype but with an internal tandem duplication of FLT3 (*FLT3-ITD*) have an inferior outcome compared to those without a *FLT3-ITD* mutation [40, 41]. In several additional studies, the presence of an *NPM1* mutation with or without the presence of a *FLT3-ITD* mutation was associated with higher complete response and event-free survival [42, 43]. Normal karyotype AML with mutations in *CEBPA* also appear to represent a subset of AML with more favorable outcome [44, 45]. Given the identification of multiple new molecular genetic abnormalities in patients with AML, an increasing number of studies have been performed to delineate the mutational frequency and prognostic implications of *IDH1/2*, *TET2*, and *DNMT3A* mutations in AML patients. The initial report of *IDH1* mutations in AML found that 8.5% of AML samples contained a mutation at amino acid R132. All mutations were associated with samples that displayed intermediate risk cytogenetics, with the large majority having normal cytogenetics. With this relatively small group of 187 samples, the investigators noted no independent prognostic value on overall survival (OS), but subgroup analysis suggested adverse effects in those patients with no *NPM1* mutations [7]. Ward et al. also assessed for mutations in a series of 87 AML samples and found that 7.7% had *IDH1* mutations. Additionally, they found that even a larger subset of samples (15.4%) displayed mutations in the *IDH2* gene. All samples with *IDH* mutations were found to have normal karyotypes, without an effect on overall survival [15].

Larger studies, based in North America and Europe, have attempted to further investigate the prevalence and prognostic implications of these mutations. A CALGB study assessed bone marrow and peripheral blood marrow samples from 358 patients with cytogenetically normal AML. The investigators reported *IDH* mutations in the third of their patients, with 14% having IDH1 mutations and 19% displaying *IDH2* mutations. AML patients with *IDH* mutations in this study tended to be younger with lower-risk disease as defined by a higher frequency of *NPM1* mutations without the presence of *FLT3-ITD* mutations. Specifically, *IDH1* mutations adversely affected disease-free survival (DFS) in this particular subgroup of patients with a favorable molecular profile. Interestingly, those patients with *IDH2* mutations achieved a lower rate of complete remission with induction therapy [12]. 

A study from The Netherlands also found a significant prevalence of *IDH* mutations in 893 samples from patients with newly diagnosed AML, with 6% and 11% having *IDH1* and *IDH2* mutations, respectively. They also reported an association with normal karyotype AML and the *NPM1* mutation. As in previous studies, no effect on OS was noted for the entire cohort of AML samples, but interestingly, in those samples *without* an *NPM1* mutation, IDH mutations were associated with an inferior event-free survival (EFS) [10]. A subsequent study from the UK found *IDH1* mutations in 8% of patient samples, using data on 1333 adult patients from the UK MRC AML10 and AML12 trials. Similar to the Dutch study, they reported a strong association with intermediate cytogenetics and the *NPM1* mutation. However, in contrast to the previous study but similar to results of the CALGB trial by Marcucci et al. [12], the investigators found that an *IDH1* mutation preferentially reduced survival in the cohort of patients *with* a coexistent *NPM1* mutation [46]. These findings were supported by large studies from France and Germany, which again found that 12–16% of patients had IDH mutations, with the mutations adversely affecting rates of CR and survival in the subgroup of patients with CN-AML and *with NPM1* mutations [11, 13]. 

However, the prognostic significance of the copresence of *IDH* and *NPM1* mutations in CN-AML continues to be uncertain. A recent large German study reported shorter EFS and rates of CR in all AML patients with an *IDH1* mutation, and specifically within the population of patients *without NPM1* mutations [14]. The same authors have recently also presented their data at the annual American Society of Hematology (ASH) 2010 meeting on 526 patients with cytogenetically normal AML and found that 28.7% of patients had *IDH* mutations. 12.9% of patients were found to have *IDH1* mutations and 15.8% had IDH2 mutations. In their survival analysis, no effect was found on OS or EFS. However, in this presentation, a trend for shorter EFS was reported in all IDH mutant patients *with* NPM1+/FLT3-ITD genotype, and, interestingly, a significant adverse effect on EFS in those with specifically IDH2 mutations in the NPM1+/FLT3-ITD-group [47]. The leukemia group from M.D. Anderson in Houston, Tex, USA, also presented their data at the ASH 2010 meeting on 358 AML patients treated with induction chemotherapy. They found that *IDH1* and *IDH2* mutations were associated with normal karyotype and *NPM1* mutations and were present in 12 (7%) and 24 (14%) patients and also found an IDH*1G105* single-nucleotide polymorphism (SNP) alteration in 24 (14%) patients. Overall, they found that 30% of patients had IDH alterations, but there was no association with achievement of CR, remission duration, OS or EFS, and IDH aberrations [48]. Caramazza et al. from the Mayo Clinic examined *IDH* mutations in 157 patients with hematologic malignancies and suggested an association with isolated trisomy 8. Eighteen IDH mutations were identified, with the majority (15) being *IDH2* mutations. Seventeen of the 18 *IDH* mutations occurred in myeloid malignancies, among which the large majority were MDS or AML. *IDH* mutations were fewer among 64 additional patients with AML or MDS without isolated trisomy 8. In MDS patients with trisomy 8 alteration, prognosis was similar between those with *IDH* mutations and those without [49]. 

### 2.2. *TET2* Mutations

*TET2*, the ten-eleven-translocation gene 2, is located in chromosome 4 at band 4q24. Delhommeau and colleagues first described the presence of *TET2* mutations in myeloid malignancies, by evaluating bone marrow samples from 320 patients with MDS, MPNs, and AML. *TET2* defects, either mutations or deletions, were discovered in 17 of 90 patients with MDS (19%), in 24 of 198 patients with MPN (12%), and in 5 of 21 patients with secondary AML (24%) [50]. Other groups have since reported a similar prevalence of TET2 mutations in myelodysplastic syndromes and other myeloid diseases [51–53]. *TET2* mutations were also found in the large majority (median of 96%) of the precursor cells in the bone marrow of patients with mutations, including CD34+ progenitor cells [54]. In addition, there has also been much interest in the role of *TET2* as a transformative mutation in myelodysplasia and MPNs. By analyzing the DNA of paired samples from patients with transformed, secondary AML, it was found that *TET2* mutations were detected most frequently after the transformation of MPNs to AML [55]. 

The pathogenic mechanism of *TET2* mutations in myeloid diseases has been an area of intense investigation. Strong evidence supports the integral role of *TET2* in demethylation of DNA and epigenetic regulation, specifically in the conversion of 5-methylcytosine to 5-hydroxymethylcytosine (5-hmC) [30, 31]. A recent intriguing study convincingly demonstrated that 5-hmC is an intermediary in the process of DNA demethylation, and that TET hydroxylases are essential mediators of this process [56]. Ko et al. further demonstrated that mutations of *TET2* lead to alteration and suppression of catalytic activity of the enzyme. They reported low DNA levels of 5-hmC in bone marrow samples taken from patients with *TET2* mutations. In addition, the depletion of *TET2* in murine models led to a suppression of differentiation of hematopoietic precursors in culture [57]. This interruption in myelopoiesis may be a necessary “hit” or alteration in the process of leukemogenesis in patients with TET-2 mutant AML.

Most recently, a variety of groups have studied the prognostic impact of *TET2 *mutations in MDS and AML. They appear to have a particularly high prevalence in chronic myelomonocytic leukemias, in which they have been found to be associated with significant monocytosis and poor outcomes [58]. Data on prognosis has also been presented in patients with MDS and AML, where there has been some controversy. Nibourel et al. sequenced sample DNA from 111 patients who had achieved CR after induction chemotherapy for de novo AML. They reported an overall incidence of 17% of *TET2 *mutations, which were associated with *NPM1* mutations but did not appear to have an impact on survival. This lack of effect on outcomes was also reported by other investigators [59, 60]. Another group from France reported that TET2 mutations were actually independently associated with significantly improved overall and progression-free survival in patients with MDS. Only a small percentage of patients (7%) in this study were reported to have secondary AML [61]. In contrast, other investigators have reported a significant adverse effect on overall survival in patients with AML [51], and a recent CALGB report of 427 patients with cytogenetically normal AML found that *TET2 *mutations were associated with a lower rate of CR and shorter disease-free and overall survival, with outcomes particularly worse for patients with favorable risk (*CEPBA* and *NPM1* mutant) disease [62]. 

Perhaps, more intriguing has been the consistent finding in recent studies that *TET2* mutations rarely cooccur with mutations affecting isocitrate dehydrogenase (*IDH1 *and* IDH2*) [60, 62]. As mentioned, the possible mechanism for aberrant methylation and leukemogenesis in IDH-mutant AML may be related to the downregulation of *α*-KG levels, on which TET2 enzymes depend for their activity [26]. Others have reported that the metabolite 2-HG, markedly elevated in samples of patients with IDH mutant AML, can also directly inhibit TET2 function [32]. Therefore, *TET2 *and* IDH* mutations may be leukemogenic through a common mechanism, that of suppression of TET2 function. They, thus, likely act as a distinct mutational class in AML with overlapping effects on DNA methylation and leukemogenesis (Figure 1).

### 2.3. *DNMT3A* Mutations

DNA methyltransferases catalyze the methylation of cytosine residues of CpG dinucleotides in DNA and are encoded by the human genes *DNMT1*, *DNMT3A*, and *DNMT3B*. *DNMT3A* mutations in AML have only been recently described [63–65] and found to be present in approximately 20–22% of patients with *de novo *AML. Interestingly, these mutations seem to be associated with intermediate-risk AML, a finding also noted with *TET2*- and *IDH*-mutant disease. In contrast, no cases of favorable risk AML contained these mutations [63]. Unlike the exclusivity of *TET2* and *IDH* mutations in recent studies of AML, *DNMT3A* mutations often cooccurred with *IDH* mutations, suggesting that these latter two mutations may not have overlapping functions in leukemogenesis. 

Somatic mutations in *DNMT3A* have been reported as nonsense, frameshift, and missense mutations throughout the open-reading frame. However, a notable recurrent mutation in *DNMT3A* has been repeatedly reported in AML [63] and MDS [66] patients as a somatic missense mutation at amino acid R882. Although one study reported a decrease in DNA methylation activity of >50% with the *DNMT3A R882 *mutant [64] in an *in vitro *methyltransferase assay, AML patient samples with *DNMT3a* mutations were not found to have altered total 5-methylcytosine content or altered patterns of methylation [63] (Figure 1). Equally important is the fact that the *DNMT3A R882A* mutation appears to occur exclusively as a heterozygous mutation suggesting a potential gain of function which may or may not require a wildtype copy of DNMT3a for altered function. Future studies examining the function of the *DNMT3A R882* mutation *in vitro* and *in vivo* in the presence of wildtype DNMT3A will hopefully shed further light on a pathogenic mechanism of *DNMT3a* mutations in AML.

*DNMT3A* mutations were subsequently noted in patients with MDS and secondary AML. Walter et al., sequencing samples from 150 patients, found that 12 harbored *DNMT3A* mutations. Similar to the trend noted in AML, the majority of mutations were at amino acid R882. These mutations were associated with worse overall survival and rapid progression to AML, although sample size was small and transplantation status was not considered [66]. The adverse impact on survival has also been reported in patients with AML. Ley and colleagues found, in their cohort, that those with *DNMT3A *mutations experienced a significantly worse median overall survival of 12.3 months as compared to 41.1 months for those without mutations. Interestingly, it appeared that *DNMT3A* mutations accounted for the majority of the adverse effect on survival seen even in patients with FLT3-ITD alterations [63]. A more recent study from China, studying patients with acute monocytic leukemias, also reported decreased overall survival and worse outcomes in those with *DNMT3A *mutations [65]. Lastly, *DNMT3a* mutations have also recently been reported in patients with primary myelofibrosis [67]. Larger studies of *DNMT3a* mutations in patients with additional MPNs will be needed to further understand the clinical and/or prognostic importance of DNMT3a mutations in MPNs.

### 2.4. *EZH2* Mutations

EZH2 is a highly conserved enzyme which serves as a histone H3 lysine 27 (H3K27) methyltransferase. Although EZH2 has been known to be overexpressed in several epithelial malignancies for some time, only in 2010 was it discovered that *EZH2* may be mutated in hematopoietic malignancies. Curiously, a recurrent mono-allelic activating mutation has been identified in EZH2 at tyrosine 641 in patients with lymphomas [68] while a series of apparent loss-of-function mutations have been found in patients with MDS and primary myelofibrosis [69–71]. 

The fact that EZH2 is altered by overexpression/increased activity in epithelial cancers and lymphomas, yet inactivated in myeloid malignancy, argues that the biologic consequences of alterations in H3K27me3 may be tissue specific. At the same time, rigorous assessment of whether *EZH2* mutations affect the abundance and/or the distribution of H3K27me3 in the chromatin of malignant versus paired normal nonmutated cells has not yet been published (Figure 1). In addition, investigation of the effects of *EZH2 *mutations on alterations in DNA methylation may be particularly important given that EZH2 physically interacts with DNA methyltransferases 1–3 [72], and data suggests that H3K27 methylation is a necessary prerequisite for DNA promoter methylation [72]*.*

From a clinical standpoint, inactivation of EZH2 by loss or mutation in MDS is enlightening as cytogenetic abnormalities of chromosome 7 which have been long recognized in MDS and AML and linked to adverse outcome [73, 74]. In fact, several studies suggest that MDS patients with *EZH2* mutations have worsened overall survival compared to those without *EZH2* mutations, regardless of gross cytogenetic findings [70, 71]. Larger studies incorporating *EZH2* mutations in light of additional genetic abnormalities will be needed to further clarify the prognostic importance of *EZH2* mutations in MDS and MPNs and are ongoing.

## 3. The Prospect of Novel Therapeutic Agents Targeting Epigenetic Modifiers in Myeloid Malignancies

### 3.1. DNA Methyltransferase Inhibitors (DNMTIs)

The first three epigenetic targeted therapeutics which have been FDA approved for use in the United States include 2 drugs targeting DNMTs (azacitidine (AZA) and decitabine (5-aza-2′-deoxycytidine)) as well as one histone deacetylase inhibitor (vorinostat). Although the use of DNMTIs has proven useful in the therapy of high-risk MDS as well as in AML, there are several questions which have lingered regarding the use of these therapies: (1) what is the ideal dose and schedule of DNMTIs? (2) what is the true mechanism of action of the nucleoside DNMTIs? (3) are there biomarkers which can be used for predicting response and/or resistance to DNMTIs? and (4) can we develop nonnucleoside direct inhibitors of DNMTs? 

Despite questions regarding use and schedule of DNMTIs, there have been several interesting new developments in this class of therapeutics. Within the original category of nucleoside analog DNMTIs, an oral formulation of AZA has recently been developed [75]. Parenteral azacitidine is approved for administration at 75 mg/m^2^ for 7 days every 28 days, and this dose is believed to result in DNA hypomethylation as well as cytotoxicity. Using several assays for DNA methylation, this dose of 5-aza has been shown to result in maximal DNA hypomethylation at approximately day 15 with gradual return of DNA methylation back to baseline around the time of next cycle [76]. Given this, use of orally administered AZA on a more frequent schedule may result in altered effects on DNA methylation and cellular cytotoxicity and holds the potential for greater therapeutic efficacy. The initial phase I trial of oral AZA on a 7 days schedule revealed that the drug is bioavailable, safe, and clinically active in patients with MDS and AML. At the 2010 ASH meeting, results of the multicenter phase I study of extended oral AZA schedules revealed that oral azacitidine on a 14- or 21-day schedule is well tolerated, with no AZA accumulation, and promising clinical responses were observed [75].

The direct cytotoxic effects of the clinically utilizing DNMTIs as well as their chemical instability have prompted continuous rationale for developing additional DNMTIs. A third nucleoside DNMTI which has been under development for some time is the cytidine analogue zebularine. Although zebularine has a similar mechanism of action as decitabine and azacitidine, resulting in the depletion of DNMTs through covalent bonding with DNMTs, zebularine has a much longer half-life making oral administration of the drug possible [77, 78]. Additionally, zebularine appears to be selectively incorporated into malignant and not normal cells admixed with tumor in at least one setting, a property not seen with decitabine or AZA [79]. Despite these properties, one limitation to the development of zebularine for clinical use has been the fact that higher concentrations of zebularine are needed to obtain similar levels of demethylation in cells in comparison with azacitidine and decitabine [78]. Further preclinical works addressing the practicality of the drug as a clinical therapeutic agent are ongoing.

In addition to the nucleoside DNMTIs, there has been considerable efforts at developing nonnucleoside targeted molecules to directly inhibit individual DNMTs. One approach has been the development of antisense oligonucleotides targeting DNMT1 for *in vivo* use. One such molecule, the phosphorothioate antisense oligonucleotide MG98, was developed based on its ability to knockdown DNMT1 expression in various model systems [80, 81]. However, phase I clinical trials of MG98 in solid and hematopoietic tumors was disappointing with very little consistent knock down of DNMT1 mRNA in patients [82, 83]. This likely resulted from inefficient intracellular uptake of MG98. 

Rational design of small molecules targeting DNMTs through noncovalent interactions with the catalytic sites of these enzymes has resulted in the development and characterization of several test compounds. The first rationally designed DNMT1 inhibitor is RG108 which was designed utilizing a three-dimensional model of the human DNMT1 catalytic pocket [84]. RG108 has comparable demethylating activity to zebularine but appears to be less active than azacitidine and decitabine. Several additional small-molecular inhibitors of DNMTs have more recently been found within the NCI open database of compounds through a similar screening approach which led to the discovery of RG108 [85]. Further preclinical characterization of all of these compounds is underway. 

The use of DNMTIs in patients with high-risk MDS and AML has proven that while durable complete remissions are possible with these drugs, responses can be quite variable with no current routinely used clinical parameter known to predict likelihood of response to therapy. With the discovery of mutations in *TET2 *in these patients and the postulated pathogenic mechanism of *TET2* mutations in MDS/AML, it has been hypothesized that *TET2*-mutated patients may have higher rates of response to DNMTIs. This has recently been suggested by a small French study of 86 patients with MDS and secondary AML. The investigators reported that those with *TET2* mutations experienced a response rate (RR) of 82% to AZA in comparison to the *TET2*-wt group, which had a significantly lower RR of 45%. However, there was no effect on survival parameters, and the study group was quite heterogeneous with few additional genetic parameters studied [86]. In contrast, other groups have found that *TET2* alterations in a similar cohort of patients may actually predict for decreased responsiveness to demethylating therapies [87]. The small number of patients included in these studies and the limited genetic characterization of the patients must be considered. Larger studies with more comprehensive genetic evaluation will be critical in determining if mutations in genetic factors suspected to be important in regulating DNA methylation (*TET2*, *IDH1/2*, and *DNMT3a* mutations, amongst others) affect response to DNMTIs.

## 4. The Prospect for Novel Histone Methyltransferase Inhibitors in Myeloid Leukemias: DOT1L Inhibition and Rational Design of Protein Methyltransferase Inhibitors

Recent discovery of the potential importance of aberrant hypermethylation of histone lysines in the pathogenesis of myeloid leukemias driven by MLL-translocations [88], NUP98-NSD1 translocations [89], and possibly *IDH1/2*-mutant disorders [32] suggests the possibly of targeting histone lysine methyltransferases in myeloid leukemias. 

 One novel and exciting prospect utilizing this therapeutic rationale is the study of DOT1L-targeted therapy for the selective treatment of MLL-translocated leukemias. In addition to the recently identified mutations in epigenetic modifiers in MDS and AML, a number of translocations disrupting the normal activity of epigenetic modifiers in myeloid malignancies have been recognized for a longer period of time (Table 1). Key amongst the frequent translocations altering the activity of an epigenetic modifiers in AML includes translocations involving *mixed lineage leukemia 1 *(*MLL1*) which occur in at least 10% of adult AML patients and >70% of infant acute leukemias. MLL1 normally serves as an histone H3 lysine 4 (H3K4) methyltransferase. MLL1 translocations result in fusion of the N terminus of MLL1 to one of >60 different translocation partners [90]. In a recent landmark survey of MLL rearrangements, 760 MLL-rearranged biopsy samples were reviewed and 104 different MLL rearrangements were found in adult and pediatric acute leukemia patients [90]. However, amongst AML patients with MLL rearrangements, 77% were accounted for by one of 7 translocations: MLL-AF9 (30.4%), MLL-AF10 (14.5%), MLL-ELL (10.9%), MLL-AF6 (10.1%), MLL-ENL (5.4%), MLL-AF17 (2.9%), and MLL-SEPT6 (2.5%). Many of these same MLL rearrangements are also common in acute lymphoblastic leukemia (ALL) along with MLL-AF4 which is the most common MLL rearrangement in ALL (accounting for 66% of MLL-rearranged ALL cases) [90]. 

Importantly, the four most frequent MLL translocations (MLL-AF4, MLL-AF9, MLL-AF10, and MLL-ENL) result in recruitment of DOT1L (disruptor of telomeric silencing 1-like) to the fusion protein and acquisition of histone 3 lysine 79 (H3K79) methyltransferase activity (Figure 1). A number of studies using both shRNA for DOT1L and conditional deletion of DOT1L have recently shown that the H3K79 methyltransferase activity is critical for leukemogenesis induced by MLL-fusion proteins [91, 92]. This has led to the concept of developing targeted therapy for DOT1L inhibition in the therapy of MLL-translocated leukemias. At the 2010 ASH meeting, investigators at Epizyme Inc. presented the initial results from *in vitro* studies of the first DOT1L inhibitor, EPZ01 [93]. EPZ01 acts as a competitive inhibitor of the cofactor S-adenosylmethionine (SAM), the universal methyl donor for all enzymatic methyltransferase reactions. Despite the ubiquity of SAM in protein methyltransferase reactions, EPZ01 is reported to have a 500-fold selectivity for DOT1L over other lysine histone and arginine methyltransferases. Consistent with this, the investigators revealed selective killing of EPZ01 for leukemia cell lines bearing MLL1 translocations over non-MLL rearranged cell lines. DOT1L-inhibition appeared to downregulate H3K79me3 abundance globally and at critical loci, serving both as proof of concept of the mechanism of activity and potentially as a biomarker of response [93]. 

As mentioned earlier, MLL rearrangements are also frequent in ALL with MLL-AF4 translocation being the most frequent MLL rearrangement in ALL [90]. Although two initial transgenic mouse models of MLL-AF4 fusion gene overexpression did not result in the development of acute leukemia, a conditional knockin model of MLL-AF4 [94] as well as a retroviral transplantation model of MLL-AF4 [95] did result in the development of ALL. Moreover, mice with overexpression of MLL-AF4 in the conditional knockin model by Krivstov et al. were clearly distinguished by increases in H3K79me3 indicating a clear link between the presence of MLL-AF4 fusion oncoprotein and acquisition of increased H3K79 methyltransferase activity. In addition, recent purification of the MLL-AF4 complex has clearly indicated binding of DOT1L to this complex [96]. Equally important, use of an shRNA against DOT1L inhibited the expression of several genes critical for MLL-AF4-mediated oncogenesis in the MLL-AF4-conditional knockin model underscoring the potential importance of DOT1L inhibition in the therapy of ALL with MLL-AF4 rearrangement [94]. 

One key question which must be further addressed in the preclinical development of DOT1L-targeted therapy is the question of potential adverse ramifications of DOT1L inhibition. Jo et al. recently reported that mice bearing conditional disruption of DOT1L-developed pancytopenia and failure of hematopoietic homeostasis revealing a critical role of DOT1L in normal hematopoiesis [91]. At the same meeting, Bernt et al. also developed a conditional deletion model of DOT1L *in vivo* using Vav-Cre technology for DOT1L deletion in the adult hematopoietic and endothelial cells but also in the germline [92]. DOT1L^−/−^ mice in this system were born at Mendelian rations and with blood counts at lower border of normal range. In addition, cardiac-specific deletion of DOT1L resulted in increased mortality in mice due to cardiac dysfunction which closely resembled human dilated cardiomyopathy [97]. Interestingly, Nguyen et al. further discovered that DOT1L is downregulated in patients with idiopathic dilated cardiomyopathy, and the cardiac phenotype in mice could be rescued by expression of dystrophin [97]. Further characterization of these mouse models and use of DOT1L inhibitors in preclinical *in vivo* testing will hopefully clarify the potential utility and safety of this very promising new therapeutic strategy.

Development of other specific inhibitors of histone methyltransferases holds promise in myeloid leukemias. The challenges for this prospect have been twofold: (1) knowledge of the genome-wide and locus-specific effects of histone modifications due to direct genetic abnormalities found in myeloid leukemia patients has been less clear for the majority of non-MLL translocated patients. For instance, loss-of-function mutations in the H3K27 methyltransferase *EZH2* has been recently found in patients with MDS and primary myelofibrosis [69–71]. At the same time, loss of the H3K27 demethylase UTX has also been suggested to occur in some of the same disorders making the rationale for targeted changes affecting H3K27 methylation hard to understand [98]. (2) In addition, from a drug development standpoint, potent and selective inhibitors of histone protein methyltransferases have only recently begun. One strategy mentioned earlier is the development of small-molecule inhibitors of the SAM-binding pocket, a universal feature of protein methyltransferase somewhat analogous to the targeting of the ATP-binding pockets of protein kinases. In fact, the first selective and potent protein methyltransferase inhibitor was recently reported using this strategy [99]. This molecule serves to inhibit the arginine methyltransferase CARM1, a protein overexpressed and thought to be important in the pathogenesis of prostate and breast carcinomas [100, 101]. Although empiric use of these medications in early-phase clinical trials has been utilized previously with some success, in-depth characterization of histone posttranslational modifications in patients with MDS/AML may shed light on rational strategy for specific histone methyltransferase inhibition as a therapeutic strategy in these disorders.

## 5. Conclusion

The exciting discovery of new genetic abnormalities in patients with myeloid malignancies holds the promise for furthering our understanding of the pathogenesis of these disorders but also in refining our risk stratification and therapeutic management of patients. As highlighted here, a series of studies have rapidly suggested that mutations in *TET2*, *IDH1/2*, and *DNMT3a* will likely refine our current prognostication of patients with AML if borne out repeatedly in large prospective trials of AML patients. Moreover, given the suggested effects of these genetic abnormalities on DNA methylation, the potential importance of these mutations on affecting response to DNMTIs will need to be more thoroughly investigated. The effects of the recurrent *DNMT3a R882* mutation will particularly need to be scrutinized given its frequency and the fact that it is always present as a heterozygous mutation. Furthering our understanding of the specific altered epigenetic marks placed by genetic abnormalities in MDS/AML patients is critical as it may result in the development of novel epigenetic targeted therapeutics. The clearest example of this currently is the exciting development of DOT1L inhibitors for MLL-translocated leukemias described here. Further characterization of DOT1L deficiency/inhibition in a variety of *in vivo* models is greatly anticipated. Moreover, development of additional protein methyltransferase inhibitors is likely forthcoming and prompts for greater understanding of the epigenetic alterations present in patients with MDS and AML.

## Figures and Tables

**Table 1:** Translocations directly affecting histone modifying enzymes or recruitment of histone modifying enzymes in patients with myeloid malignancies.

Gene	Effects of translocation on histone posttranslational modifications
MLL1	MLL1 normally serves as an H3K4 methyltransferase. MLL-AF4, MLL-AF9, MLL-AF10, and MLL-ENL translocations result in loss of the SET domain and recruitment of DOT1L binding resulting in acquisition of H3K79 methyltransferase activity.

CBP	The histone acetyltransferase CBP has been reported to undergo translocation with MOZ in AML. This results in the disruption of CBP's normal acetyltransferase activity and also in recruitment of CBP to MOZ-regulated gene promoters. MOZ also contains a putative acetyltransferase domain which may be affected in this translocation. CBP is also an occasional translocation partner with MLL1.

NSD1	The H3K36 methyltransferase NSD1 has been rarely reported to undergo translocation with NUP98 in AML. This translocation does not abrogate H3K36 methyltransferase activity of NSD1 but rather promotes aberrant H3K36 methylation at specific loci which promotes leukemogenesis.

P300	The histone acetyltransferase p300 is an occasional translocation partner with MLL1 in AML. This translocation preserves the majority of the coding sequence of p300, and the direct transcriptional and histone effects of this translocation are not well characterized. Interesting p300- and CBP-MLL translocations appear to be significantly associated with therapy-related AML rather than *de novo *AML suggesting a potential difference in the pathogenesis of these 2 subtypes of AML.

AML1	Translocations involving AML1 are characteristic of a proportion of patients with core-binding factor leukemias. Normally the C terminus of AML1 interacts with the histone acetyltransferase p300 and recruits p300 to specific loci bound by the N terminus of AML1. However, in the common translocation t(8;21)(q22;q22), the C terminus of AML1 is lost and replaced with the C terminus of ETO which attracts a corepressor complex with histone deacetylase activity (N-CoR/Sin3/HDAC complex).

RAR*α*	The characteristic translocation of acute promyelocytic leukemia, t(15;17)(q21;q21) fuses PML with RAR*α*. Recently it has been demonstrated that one of the critical aspects of PML-RAR*α*-induced oncogenesis is aberrant downregulation of histone H3 acetylation by the PML-RAR*α* fusion protein. Normally, in the presence of its ligand retinoic acid, RAR*α* functions as a transcriptional activator. However, when ligand is not present, RARa functions as a transcriptional repressor through recruitment of HDACs. The PML-RAR*α* fusion protein results in constitutive HDAC activity and aberrant target gene repression. Pharmacologic doses of ATRA appear to greatly increase histone H3 acetylation, and this, in part, serves to reverse some of the oncogenic effects of the PML-RAR*α* fusion protein.
